# Supplementary calcium ameliorates ammonium toxicity by improving water status in agriculturally important species

**DOI:** 10.1093/aobpla/plv105

**Published:** 2015-09-02

**Authors:** Elvia Hernández-Gómez, Luis A. Valdez-Aguilar, Donita L. Cartmill, Andrew D. Cartmill, Irán Alia-Tajacal

**Affiliations:** 1 Programa de Agricultura Protegida, Universidad Autónoma Agraria Antonio Narro, Calzada Antonio Narro 1923, Buenavista, Saltillo, Coahuila 25315, México; 2 School of Agriculture, University of Wisconsin–Platteville, 1 University Plaza, Platteville, WI 53818, USA; 3 Facultad de Ciencias Agropecuarias, Universidad Autónoma del Estado de Morelos, Avenida Universidad 1001, Cuernavaca, Morelos 62209, México

**Keywords:** Antioxidant activity, electrolyte leakage, lipid peroxidation, nutrient imbalance, photosynthesis rate, root hydraulic conductance, stress tolerance

## Abstract

Fertilization of agricultural plants with ammonium $\left(\text{N}{{\text{H}}_{4}}^{+}\right)$ is often desirable because it is less susceptible to leaching than nitrate $(\text{N}{{\text{O}}_{3}}^{-}),$ reducing environmental pollution, risk to human health and economic loss. However, a number of important agricultural species exhibit a reduction in growth when fertilized with $\text{N}{{\text{H}}_{4}}^{+},$ and increasing the tolerance to $\text{N}{{\text{H}}_{4}}^{+}$ may be of importance for the establishment of sustainable agricultural systems. The present study explored the feasibility of using calcium (Ca) to increase the tolerance of bell pepper (*Capsicum annuum*) to $\text{N}{{\text{H}}_{4}}^{+}$ fertilization. Although $\text{N}{{\text{H}}_{4}}^{+}$ at proportions ≥25 % of total nitrogen (N) decreased leaf dry mass (DM), supplementary Ca ameliorated this decrease. Increasing $\text{N}{{\text{H}}_{4}}^{+}$ resulted in decreased root hydraulic conductance (*L*_o_) and root water content (RWC), suggesting that water uptake by roots was impaired. The $\text{N}{{\text{H}}_{4}}^{+}$-induced reductions in *L*_o_ and RWC were mitigated by supplementary Ca. Ammonium induced increased damage to the cell membranes through lipid peroxidation, causing increased electrolyte leakage; Ca did not reduce lipid peroxidation and resulted in increased electrolyte leakage, suggesting that the beneficial effects of Ca on the tolerance to $\text{N}{{\text{H}}_{4}}^{+}$ may be more of a reflection on its effect on the water status of the plant. Bell pepper plants that received $\text{N}{{\text{O}}_{3}}^{-}N$ had a low concentration of $\text{N}{{\text{H}}_{4}}^{+}$ in the roots but a high concentration in the leaves, probably due to the high nitrate reductase activity observed. Ammonium nutrition depressed the uptake of potassium, Ca and magnesium, while increasing that of phosphorus. The results obtained in the present study indicate that $\text{N}{{\text{H}}_{4}}^{+}$ caused growth reduction, nutrient imbalance, membrane integrity impairment, increased activity of antioxidant enzymes and affected water relations. Supplementary Ca partially restored growth of leaves by improving root *L*_o_ and water relations, and our results suggest that it may be used as a tool to increase the tolerance to $\text{N}{{\text{H}}_{4}}^{+}$ fertilization.

## Abstract (precis)

Fertilization of plants with ammonium is highly desirable because it is less susceptible to leaching than nitrate, with associated reductions in environmental pollution and risk to human health; however, important agricultural species exhibit a reduction in growth when fertilized with high levels of ammonium. The present study proved that tolerance to ammonium can be increased in bell pepper plants when calcium is incorporated at higher concentrations; calcium should thus be integrated into crop management to enhance the tolerance of agricultural plants to excess ammonium.

## Introduction

Nitrate $\left(\text{N}{{\text{O}}_{3}}^{-}\right)$ and ammonium $(N{{\text{H}}_{4}}^{+})$ are the predominant forms in which nitrogen (N), a critical nutrient for the growth of agricultural species (Berger *et al*. 2013), is absorbed; nonetheless, the growth of cultivated plants may be affected by the predominant form of N present in the soil or nutrient solution. The use of $\text{N}{{\text{H}}_{4}}^{+}$ as the N source for crop production is particularly attractive when compared with $\text{N}{{\text{O}}_{3}}^{-}$ as it is less susceptible to leaching from the soil profile (Fernández-Escobar *et al*. 2004), potentially increasing N use efficiency and reducing environmental pollution and the risk of human diseases, such as methemoglobinemia, cancers of the digestive tract, thyroid problems and diabetes (Knobeloch *et al*. 2000). Economic issues must also be considered as the production of $\text{N}{{\text{H}}_{4}}^{+}based$ fertilizers is less expensive than that of $\text{N}{{\text{O}}_{3}}^{-}based$ fertilizers because a higher energy input is needed to oxidize $\text{N}{{\text{H}}_{4}}^{+}$ into $\text{N}{{\text{O}}_{3}}^{-}$ (Havlin *et al*. 2013).

Plants may also benefit from $\text{N}{{\text{H}}_{4}}^{+}$ nutrition when provided at limited concentrations as its assimilation represents a lower energy cost due to its redox state, eliminating the requirement for reduction (Britto and Kronzucker 2002), which may result in increased plant growth and yield. However, high concentration of $\text{N}{{\text{H}}_{4}}^{+}$ may be toxic to some agricultural species (Borgognone *et al*. 2013), mainly due to a depletion of carbon supply caused by damage to the chloroplast ultrastructure (Takács and Técsi 1992), decreased photosynthesis rate (Setién *et al*. 2013) and an energy-demanding transmembrane $\text{N}{{\text{H}}_{4}}^{+}$ cycle in order to maintain low intracellular $\text{N}{{\text{H}}_{4}}^{+}$ concentrations (Britto *et al*. 2001). Other plant responses to high concentration of $\text{N}{{\text{H}}_{4}}^{+}$ include increased intracellular pH (Bittsánszky *et al*. 2015), damage to the cell membrane permeability (M'rah-Helali *et al*. 2010) and decrease in the uptake of essential cations such as potassium (K), calcium (Ca) and magnesium (Mg) (Hawkesford *et al*. 2012).

Ammonium at high concentrations induce a reduction in K uptake in several species; however, enhancing the external supply of K has been proposed as a tool for the alleviation of $\text{N}{{\text{H}}_{4}}^{+}$ toxicity (ten Hoopen *et al*. 2010). Potassium ameliorates $\text{N}{{\text{H}}_{4}}^{+}$ toxicity by reducing the free $\text{N}{{\text{H}}_{4}}^{+}$ concentration in the shoot and root tissues and by reducing the futile cycling of $\text{N}{{\text{H}}_{4}}^{+}$ at the plasma membrane (Balkos *et al*. 2010). Calcium concentration in plant tissues is reduced in cucumber (*Cucumis sativus*) (Roosta *et al*. 2009) and arabidopsis (*Arabidopsis thaliana*) plants (M'rah-Helali *et al*. 2010) grown at high concentration of $\text{N}{{\text{H}}_{4}}^{+},$ while tomato (*Solanum lycopersicum*) exhibits increased incidence of blossom end rot (a Ca-related disorder) (Borgognone *et al*. 2013). Improving plant performance through optimization of $\text{N}{{\text{H}}_{4}}^{+}$ nutrition and the use of supplementary levels of K, and other cations such as Ca, may be of substantial agronomic significance in global crop production (Balkos *et al*. 2010); however, little research has been conducted to elucidate the effect of supplementary concentration of Ca on the response of plants to $\text{N}{{\text{H}}_{4}}^{+}$ toxicity.

The objective of the present study was to determine the effect of $\text{N}{{\text{H}}_{4}}^{+}$ nutrition on growth, photosynthetic parameters, root hydraulic conductance (*L*_o_), leaf water potential (*ψ*_w_), antioxidant enzymes, nitrate reductase activity (NRA) and the nutrient status of *Capsicum annuum* cv. Darsena (bell pepper) plants in soilless culture, a species particularly sensitive to $\text{N}{{\text{H}}_{4}}^{+}$ and widely cultivated in protected agriculture and soilless systems in the southern portion of North America, and to explore the feasibility of using Ca to increase the tolerance to high $\text{N}{{\text{H}}_{4}}^{+}$ concentration.

## Methods

### Cultural conditions and plant material

The study was conducted under greenhouse conditions in Northeast México (25°27′LN, 101°02′LW, 1610 m above sea level); average minimum/maximum temperature and relative humidity for experiment duration were 28 °C/15 °C and 45 %/75 %, respectively, and average photosynthetically active radiation (PAR) measured at solar noon was 501 µmol m^−2^ s^−1^.

Thirty-three-day-old bell pepper plants (15 cm height) were transplanted to a 39-L rigid plastic container with a drainage hole for retrieval of the nutrient solution. The container was filled with horticultural grade perlite [33 % (v/v) water holding capacity, 64 % air-filled pore space and 0.25 g cm^−3^ apparent density], with each container being considered as an experimental unit.

### Nutrient solutions

Nutrient solutions with 14 meq L^−1^ total N were prepared with distilled water and varying proportions of $\text{N}{{\text{H}}_{4}}^{+}:$ 0 % (control), 25% and 50 %; the remaining N was applied as $\text{N}{{\text{O}}_{3}}^{-}.$ Potassium, phosphorus (P), Ca, Mg and sulfate were supplied at 6, 1, 10, 4 and 4 meq L^−1^, respectively. Nutrient solution with 50 % of N as $\text{N}{{\text{H}}_{4}}^{+}$ was prepared with two concentrations of Ca: 9 and 14 meq L^−1^. The pH of the nutrient solutions ranged from 5.8 to 6.0 and electrical conductivity (EC) from 2.0 to 2.4 dS m^−1^. Fertigation was applied through a drip irrigation system (six emitters per experimental unit), designed to collect the leachate for reuse. Plants were irrigated for 10 min (∼3.6 L) six times a day. Evapotranspirated water was replenished daily to each stock tank of nutrient solution with distilled water and the nutrient solutions were replaced every other day.

### Gas exchange parameters and leaf water potential

Net photosynthetic rate, transpiration rate and stomatal conductance were measured (LI-COR Inc., LI-6200, Lincoln, NE, USA) at noon 40 days after planting on young leaves (the third fully developed leaf from top to bottom). Average PAR, CO_2_ concentration and temperature were maintained at 400 µmol m^−2^ s^−1^, 355 p.p.m. and 25.5 °C, respectively. Three measurements on each leaf from one plant per experimental unit were recorded. Water potential of two young leaves per plant was measured 30 days after planting (Scholander Pressure Chamber, Soil Moisture Equipment Corp., Santa Barbara, CA, USA).

### Assessment of plant growth and nutrient status

Growth measurements were recorded at harvest (67 days after planting), including leaf area (LI-COR Inc., LI-3100) and dry mass (DM) of previously washed and dried stems, leaves and roots. Shoot and root water content (RWC) was calculated with the difference in fresh mass (FM) and DM {water content = [(shoot or root FM − shoot or root DM)/shoot or root DM] × 100}.

Concentration of N forms ($\text{N}{{\text{O}}_{3}}^{-}N,$ $\text{N}{{\text{H}}_{4}}^{+}N$ and total N) was determined in roots and leaves of plants sampled at experiment termination. Samples were rinsed (2×) in distilled water, bagged and dried (75 °C for 72 h), and the dried material ground to pass a 40-mesh screen (Tekmar, model A-10, Cole-Parmer Instrument, Chicago, IL, USA). Nitrate and $\text{N}{{\text{H}}_{4}}^{+}$ concentration were determined following Cataldo's (Cataldo *et al*. 1975) and Nessler's (Krug *et al*. 1979) methods. Total N was determined with the Kjeldhal's procedure. Phosphorus, K, Ca and Mg were determined in digested leaf and root tissues (2 : 1 mixture of H_2_SO_4_ : HClO_4_ and 2 mL of 30 % H_2_O_2_) with inductively coupled plasma emission spectrometer (ICP-AES, model Liberty, Varian, Santa Clara, CA, USA).

### Lipid peroxidation, antioxidant enzymes and NRA

Lipid peroxidation was measured in leaves sampled 60 days after planting by quantifying the concentration of malondialdehyde using the thiobarbituric acid method (Kuk *et al*. 2003), whereas the superoxide dismutase (SOD) and ascorbate peroxidase (APX) activities were determined according to Bonnet *et al*. (2000) and Kuk *et al*. (2003), respectively.

Nitrate reductase activity was determined 60 days after planting using the procedure described by Ventura *et al*. (2010) with minor modifications. Briefly, fresh leaf samples (0.25 g) were collected at noon and vacuum infiltrated (pressure = 50 cmHg) for 1 min in a mixture containing 0.1 M KNO_3_ and 0.1 % *n*-propanol in 50 mM KH_2_PO_4_ buffer (pH 7.0), in 1 : 20 (w : v) ratio. The reaction was allowed to proceed for 40 min at 30 °C in the dark. Samples were collected at the beginning and end of the assay, and the level of nitrite was determined with a spectrophotometer (Shimadzu UV—150-02) after the addition of 1 % sulfanilamide in 3 M HCl and 0.02 % *N*-1-naphthyl ethylenediamine mixture 1 : 1 (v : v) at 540 nm.

### Electrolyte leakage and root hydraulic conductance

Electrolyte leakage was measured in 10 young leaf segments (1 cm^2^) at 30 and 60 days after planting. The leaf segments were washed with distilled water to remove surface contamination and placed in stoppered vials containing 10 mL of distilled water. The vials were placed in a shaker at room temperature (27 °C) for 24 h at 100 r.p.m. Electrical conductivity (EC_1_) of the bathing solution was read after the incubation period and then the samples were placed in an autoclave at 120 °C for 20 min; EC_2_ was measured after cooling the bathing solution. Electrolyte leakage was calculated using the formula: 100 × (EC_1_/EC_2_).

Root *L*_o_ was measured 40 and 62 days after planting using a modified procedure as described by Joly (1989). Each plant was placed into a 100-mL beaker with distilled water. Shoots of plants were removed 1 cm above the root insertion and enclosed in a pressure chamber. A rubber tube was attached to the stump and protruded through a rubber gasket. The pressure in the chamber was increased gradually to 0.8 MPa and the sap flow captured in a glass vial for 30 min and weighed. The flow rate of sap exudate was determined as the average exudate weight per second (mg s^−1^), and *L*_o_ was estimated as the exudate flow rate versus applied pressure per gram of root (mg g^−1^ s^−1^ MPa^−1^).

### Statistical design

Six replicates of each experimental unit (container with six plants) were distributed in a complete randomized block design. Significant effects of $\text{N}{{\text{H}}_{4}}^{+}$ and Ca were determined using ANOVA and Duncan's multiple mean comparison test (*P*< 0.05) with SAS (SAS v. 8.0, SAS Institute).

## Results

Increasing $\text{N}{{\text{H}}_{4}}^{+}$ in the nutrient solution reduced leaf area and DM of stems and leaves, but increased DM of roots when $\text{N}{{\text{H}}_{4}}^{+}$ was at 50 % of total N (Table 1). Supplementary Ca at 14 meq L^−1^ partially reversed the toxicity in plants irrigated with 50 % of total N as $\text{N}{{\text{H}}_{4}}^{+}$ when compared with Ca at 9 meq L^−1^ as leaf DM was higher than that of plants with Ca at 9 meq L^−1^.

**Table 1.** Growth responses of bell pepper (*C. annuum* cv. Dársena) plants grown in perlite and irrigated with nutrient solutions of varying ammonium (NH_4_^+^) proportions and supplementary Ca. Total N concentration was constant at 14 meq L^−1^. Degrees of freedom for numerator = 3; degrees of freedom for denominator = 15. Means within the same column followed by the same letter are not significantly different according to Duncan's multiple range test (*P* < 0.05).

$\text{N}{{\text{H}}_{4}}^{+}$ (%)	Ca (meq L^−1^)	Leaf area (cm^2^ plant^−1^)	Stem DM (g plant^−1^)	Leaf DM (g plant^−1^)	Shoot DM (g plant^−1^)	Root DM (g plant^−1^)
0	9	1056a	3.21a	3.29a	6.50a	3.07bc
25	9	503b	1.22c	1.61c	2.83c	2.75c
50	9	564b	1.87b	2.02c	3.90bc	3.87a
50	14	604b	1.63bc	2.56b	4.20b	3.52ab
ANOVA	*F* ratio	17.86	21.30	17.23	21.30	4.88
		*P*< 0.001	*P*< 0.001	*P*< 0.001	*P*< 0.001	*P*< 0.015

Photosynthesis, transpiration rate and stomatal conductance were unaffected by the proportion of $\text{N}{{\text{H}}_{4}}^{+}$ in the nutrient solution (Table 2). Leaf *ψ*_w_ and RWC decreased as the proportion of $\text{N}{{\text{H}}_{4}}^{+}$ increased in the nutrient solution (Table 3); however, in plants treated with solutions containing 50 % of total N as $\text{N}{{\text{H}}_{4}}^{+},$ supplementary Ca partially restored the water status of plants. Shoot water content was not affected by $\text{N}{{\text{H}}_{4}}^{+}$ or Ca concentration (Table 3). Root *L*_o_ was decreased by increasing the proportion of $\text{N}{{\text{H}}_{4}}^{+}$ in the nutrient solution, but in plants irrigated with solutions containing 50 % of total N as $\text{N}{{\text{H}}_{4}}^{+}$ (Table 3), supplementary Ca increased water conductance.

**Table 2.** Gas exchange parameters in bell pepper (*C. annuum* cv. Dársena) plants grown in perlite and irrigated with nutrient solutions of varying ammonium (NH_4_^+^) proportions and supplementary Ca. Total N concentration was constant at 14 meq L^−1^. Degrees of freedom for numerator = 3; degrees of freedom for denominator = 15.

$\text{N}{{\text{H}}_{4}}^{+}$ (%)	Ca (meq L^−1^)	Net photosynthesis (µmol CO_2_ m^−2^ s^−1^)	Transpiration rate (mmol m^−2^ s^−1^)	Stomatal conductance (mmol m^−2^ s^−1^)
0	9	9.03	7.67	0.64
25	9	8.98	8.59	0.75
50	9	9.72	7.41	0.63
50	14	9.43	7.38	0.56
ANOVA	*F* ratio	1.19	1.67	2.24
		*P*< 0.347	*P*< 0.216	*P*< 0.126

**Table 3.** Water relations in bell pepper (*C. annuum* cv. Dársena) plants grown in perlite and irrigated with nutrient solutions of varying ammonium (NH_4_^+^) proportions and supplementary Ca. Total N concentration was constant at 14 meq L^−1^. dap, days after planting. Degrees of freedom for numerator = 3; degrees of freedom for denominator = 15. Means within the same column followed by the same letter are not significantly different according to Duncan's multiple range test (*P* < 0.05).

$\text{N}{{\text{H}}_{4}}^{+}$ (%)	Ca (meq L^−1^)	Shoot water content (%)	RWC (%)	Leaf *ψ*_w_ (MPa)	Root hydraulic conductance (mg g^−1^ s^−1^ MPa^−1^)	
					40 dap	60 dap
0	9	87.6	89.7a	−0.138a	12.09a	18.74a
25	9	87.9	84.8bc	−0.215ab	10.54ab	7.44c
50	9	87.3	84.9c	−0.428c	5.25c	4.56c
50	14	87.1	86.5b	−0.266b	8.86b	11.32b
ANOVA	*F* ratio	0.55	7.93	16.33	14.64	26.91
		*P*= 0.656	*P*< 0.001	*P*< 0.001	*P*< 0.001	*P*< 0.001

Electrolyte leakage was unaffected by the proportion of $\text{N}{{\text{H}}_{4}}^{+}$ at 30 days after planting; however, for the measurement conducted 60 days after planting, an increase in electrolyte loss was detected in plants with $\text{N}{{\text{H}}_{4}}^{+}$ at 50 % of total N (Table 4). Supplementary Ca was associated with a significant increase in electrolyte leakage in the 30- and 60-day measurements (Table 4). Increasing $\text{N}{{\text{H}}_{4}}^{+}$ caused an increase in lipid peroxidation and the activities of APX and SOD (Table 4), but reduced that of NRA (Table 5). However, when Ca was provided at higher concentration, the activity of the SOD was significantly decreased (Table 4).

**Table 4.** Effect of varying ammonium (NH_4_^+^) proportions and supplementary Ca on lipid peroxidation, electrolyte leakage, SOD and APX activities in leaves of bell pepper (*C. annuum* cv. Dársena) plants grown in perlite. Total N concentration was constant at 14 meq L^−1^. dap, days after planting. Means within the same column followed by the same letter are not significantly different according to Duncan's multiple range test (*P* < 0.05).

$\text{N}{{\text{H}}_{4}}^{+}$ (%)	Ca (meq L^−1^)	Lipid peroxidation (µm g^−1^ FM)	Electrolyte leakage (%)		SOD activity (U g^−1^)	APX activity (U g^−1^)
			30 dap	60 dap		
0	9	9.23b	15.9b	13.9b	69.6b	1109b
25	9	14.61a	17.2b	14.8b	103.2a	1430ab
50	9	11.06ab	17.8b	15.4ab	86.4ab	1470a
50	14	11.66ab	27.2a	17.3a	72.2b	1480a
ANOVA	*F* ratio	3.16	9.82	4.44	7.04	3.41
	Degrees of freedom numerator, denominator	3, 18	3, 15	3, 15	3, 15	3, 18
		*P*< 0.050	*P*< 0.001	*P*< 0.020	*P*< 0.004	*P*< 0.040

**Table 5.** Total N, ammonium (NH_4_^+^) and nitrate (NH_4_^+^) concentration (mmol kg^−1^) and NRA (nmol NO_2_ g^−1^ fresh mass h^−1^) in bell pepper (*C. annuum* cv. Dársena) plants grown in perlite and irrigated with nutrient solutions of varying NH_4_^+^ proportions and supplementary Ca. Total N concentration was constant at 14 meq L^−1^. Means within the same column followed by the same letter are not significantly different according to Duncan's multiple range test (*P* < 0.05).

$\text{N}{{\text{H}}_{4}}^{+}$ (%)	Ca (meq L^−1^)	NRA	$\text{N}{{\text{H}}_{4}}^{+}$ concentration		$\text{N}{{\text{O}}_{3}}^{-}$ concentration		Total N concentration	
			Leaf	Root	Leaf	Root	Leaf	Root
0	9	27 927a	233.0b	96.9c	108.70a	87.1ab	1284bc	1503b
25	9	25 707b	220.6b	207.6a	105.65a	102.3a	1187c	1579b
50	9	26 098b	211.1b	177.3b	39.91b	60.6bc	1388b	1492b
50	14	26 652ab	329.0a	221.8a	55.80b	40.5c	1907a	1856a
ANOVA	*F* ratio	3.10	14.63	53.14	20.69	4.97	52.98	8.77
	Degrees of freedom numerator, denominator	3, 21	3, 15	3, 15	3, 15	3, 15	3, 15	3, 15
		*P*< 0.049	*P*< 0.001	*P*< 0.001	*P*< 0.001	*P*< 0.014	*P*< 0.001	*P*< 0.001

Increasing proportions of $\text{N}{{\text{H}}_{4}}^{+}$ had no effect on $\text{N}{{\text{H}}_{4}}^{+}$ in the leaf tissue, while the root tissues exhibited increased $\text{N}{{\text{H}}_{4}}^{+}$ concentration (Table 5). Nitrate concentration was significantly decreased in both leaf and root tissues when 50 % of total N was provided as $\text{N}{{\text{H}}_{4}}^{+}$ (Table 5). Total leaf N concentration was increased as the proportion of $\text{N}{{\text{H}}_{4}}^{+}$ in the nutrient solution was increased, but root N concentration was unaffected (Table 5). Supplementary Ca was associated with a significant increase in total N and $\text{N}{{\text{H}}_{4}}^{+}$ concentrations in leaves and roots of plants irrigated with 50 % of the proportion of N in the nutrient solution provided as $\text{N}{{\text{H}}_{4}}^{+}$ (Table 5). Increasing $\text{N}{{\text{H}}_{4}}^{+}$ in the nutrient solution caused a decrease in leaf K, Ca and Mg concentrations. Phosphorus concentration in the leaf increased with increasing $\text{N}{{\text{H}}_{4}}^{+}$ in the nutrient solution (Table 6). Increasing $\text{N}{{\text{H}}_{4}}^{+}$ in the nutrient solution caused a decrease in root Ca and Mg concentration. Supplementary Ca partially counteracted the decreasing effect of $\text{N}{{\text{H}}_{4}}^{+}$ as leaf Mg and root Ca increased (Table 6).

**Table 6.** Potassium, Ca, Mg and P concentrations in leaves and roots of bell pepper (*C. annuum* cv. Dársena) plants grown in perlite and irrigated with nutrient solutions of varying ammonium (NH_4_^+^) proportions and supplementary Ca. Total N concentration was constant at 14 meq L^−1^. Degrees of freedom for numerator = 3; degrees of freedom for denominator = 15. Means within the same column followed by the same letter are not significantly different according to Duncan's multiple range test (*P* < 0.05).

$\text{N}{{\text{H}}_{4}}^{+}$ (%)	Ca (meq L^−1^)	K (mmol kg^−1^)		Ca (mmol kg^−1^)		Mg (mmol kg^−1^)		P (mmol kg^−1^)	
		Leaf	Root	Leaf	Root	Leaf	Root	Leaf	Root
0	9	1471a	943	170a	388a	232a	211a	34.8c	64.2
25	9	1273b	1007	110b	200b	139c	101b	57.9b	64.9
50	9	1316b	1143	114b	268b	190b	134b	64.5ab	54.5
50	14	1290b	1095	120b	361a	148c	121b	74.3a	77.4
ANOVA	*F* ratio	6.12	1.34	25.36	10.75	33.16	14.06	24.90	3.11
		*P*< 0.006	*P*= 0.332	*P*< 0.001	*P*< 0.003	*P*< 0.001	*P*< 0.001	*P*< 0.001	*P*= 0.081

## Discussion

Bell pepper plants were sensitive to $\text{N}{{\text{H}}_{4}}^{+}$ proportions ≥25 %, being the shoot more affected than the roots as the stems and leaves exhibited up to a 62 and 51 % decrease in DM, respectively; in the roots, plants that received 25 % of N as $\text{N}{{\text{H}}_{4}}^{+}$ showed a 26 % decrease, whereas in plants with 50 % $\text{N}{{\text{H}}_{4}}^{+},$ root growth was enhanced by 26 %. The more pronounced effect on the shoots than on the roots has been reported in species sensitive to $\text{N}{{\text{H}}_{4}}^{+}$ (M'rah-Helali *et al*. 2010; Borgognone *et al*. 2013).

Calcium is reported to increase the tolerance of plants to abiotic stresses by regulating the response reactions and developmental processes (Steinhorst and Kudla 2013). Kaya *et al*. (2002) and Tuna *et al*. (2007) reported that increased Ca concentrations resulted in enhanced growth and yield of strawberry (*Fragaria*×*ananassa*) and tomato plants exposed to high salinity; however, to our knowledge, there is no information as to the potential role of Ca in enhancing the tolerance of agricultural species to high $\text{N}{{\text{H}}_{4}}^{+}$ stress. The present study demonstrates that supplementary Ca ameliorated to some extent the stress caused by high $\text{N}{{\text{H}}_{4}}^{+}$ proportions as suggested by the higher leaf DM than that of plants with typical Ca concentrations.

The observed reduction in leaf *ψ*_w_, leaf expansion and shoot growth when $\text{N}{{\text{H}}_{4}}^{+}$ was >25 % may have been due to affected water transport to the shoot as demonstrated by the decrease in root *L*_o_ and RWC. Supplementary Ca improved to some extent root *L*_o_, resulting in increased RWC and leaf *ψ*_w_, and partially restored leaf DM accumulation. As *L*_o_ is largely determined by the activity of aquaporins (Li *et al*. 2015), probably the level of $\text{N}{{\text{H}}_{4}}^{+}$ used in the present study may have affected these water root channels, whereas Ca could have played a role in restoring it. Gao *et al*. (2010) reported that aquaporins activity in rice was higher when seedlings were supplied with N as $\text{N}{{\text{H}}_{4}}^{+}$ and indicated that the increase in *L*_o_ may be a consequence of the effect of $\text{N}{{\text{H}}_{4}}^{+}$ on root morphology (less development in the Casparian strip), growth, number of tips and surface area. However, our results showed that bell pepper plants responded differently and we have no evidence of modifications in root morphology (root DM of $\text{N}{{\text{O}}_{3}}^{-}fed$ plants was comparable with that of plants irrigated with 50 % of total N as $\text{N}{{\text{H}}_{4}}^{+}$), suggesting that the increase in *L*_o_ may be the effect of the supplementary Ca.

Surprisingly, the restoring effect of Ca on *L*_o_ did not affect gas exchange parameters. However, our data showed that the decrease in transpiration rate and stomatal conductance was associated with an increase in root Ca and N concentration. Rothwell and Dodd (2014) provided evidence that shows that Ca added to the rhizosphere can decrease stomatal conductance and photosynthesis in beans (*Phaseoulus vulgaris*) and pea (*Pisum sativum*), resulting in increased xylem sap Ca concentration in beans. However, the higher Ca concentration in the roots observed in our study in plants with supplementary Ca suggests that Ca translocation rate was maintained as in control plants as leaf Ca concentration was not increased, which suggests that this nutrient was not affecting gas exchange parameters. Nonetheless, the decrease in stomatal conductance in plants supplemented with Ca was associated with an increase in total N in leaves, which in turn was associated with a significant increase in leaf $\text{N}{{\text{H}}_{4}}^{+}$ and $\text{N}{{\text{O}}_{3}}^{-}$ concentrations. Similar effects of N nutrition of gas exchange parameters were reported by Hu *et al*. (2015) as stomatal conductance in Chinese cabbage (*Brassica pekinensis*) was decreased at proportions of 25 % of N in $\text{N}{{\text{H}}_{4}}^{+}$ form, compared with plants provided with lower $\text{N}{{\text{H}}_{4}}^{+},$ and by Du *et al*. (2015), that reported similar deleterious effects in $\text{N}{{\text{H}}_{4}}^{+}fed$ tea plants (*Camellia sinensis*) compared with $\text{N}{{\text{O}}_{3}}^{-}fed$ plants. Therefore, the no relationship between stomatal conductance and the increased root hydraulic conductance in plants supplemented with increased Ca could have been related to the increase in leaf and root $\text{N}{{\text{H}}_{4}}^{+}$ and total N concentration.

Abiotic and biotic stresses are reported to primarily target the cell membranes, so that the maintenance of the integrity and stability of the plasmalemma are of foremost importance to stress tolerance (Bajji *et al*. 2002). In the present study, bell pepper plants exhibited increased damage in cell membranes as suggested by the higher lipid peroxidation observed when N was provided as $\text{N}{{\text{H}}_{4}}^{+},$ which in turn would explain the increase in electrolyte leakage as it has also been linked with impaired membrane stability (Bajji *et al*. 2002; Tuna *et al*. 2007). However, the supplementary concentration of Ca used in the present study did not reduce lipid peroxidation and resulted in increased electrolyte leakage, which may have been the result of $\text{N}{{\text{H}}_{4}}^{+}$ leakage, in addition to other ions such as K (Demidchik *et al*. 2014), as its concentration was markedly increased in leaves of plants with 50 % $\text{N}{{\text{H}}_{4}}^{+}$ at normal and supplementary Ca concentrations.

The potential damage of reactive oxygen species (ROS) may be counteracted by increasing the activity of a group of antioxidant compounds that protect the cell membranes (Domínguez-Valdivia *et al*. 2008). In the present study, the damage to cell membranes caused by increasing $\text{N}{{\text{H}}_{4}}^{+}$ resulted in increased SOD (+42 %) and APX (+32 %) activities, suggesting that bell pepper plants attempted to detoxify ROS. However, under high $\text{N}{{\text{H}}_{4}}^{+},$ supplementary Ca was associated with a reduction in SOD activity, suggesting that ROS were causing less damage. Calcium has been associated with increased tolerance to stress by alleviating the damage caused by ROS to cell membranes in tomato plants exposed to salinity (Tuna *et al*. 2007).

Bell pepper plants that received exclusively N in $\text{N}{{\text{O}}_{3}}^{-}$ form exhibited a low concentration of $\text{N}{{\text{H}}_{4}}^{+}$ in the roots but a high concentration in the leaves, probably associated with the high reduction rate of $\text{N}{{\text{O}}_{3}}^{-}$ as a result of the high NRA activity observed in the leaves. The decreased leaf and root $\text{N}{{\text{O}}_{3}}^{-}$ concentration observed may have been caused by the lower availability of $\text{N}{{\text{O}}_{3}}^{-}$ when external $\text{N}{{\text{H}}_{4}}^{+}$ was increased and due to its rapid reduction, as suggested by the limited effect observed on NRA activity under high $\text{N}{{\text{H}}_{4}}^{+}.$

Leaf and root $\text{N}{{\text{H}}_{4}}^{+}$ concentrations were not increased even when external $\text{N}{{\text{H}}_{4}}^{+}$ was at 50 %, suggesting that bell pepper plants may regulate internal $\text{N}{{\text{H}}_{4}}^{+}$ content, probably through efflux mechanisms across the plasma membrane (Britto *et al*. 2001). The higher leaf and root $\text{N}{{\text{H}}_{4}}^{+}$ concentrations when plants were exposed to high $\text{N}{{\text{H}}_{4}}^{+}$ and higher concentrations of Ca suggest that bell pepper was able to take up higher amounts of N in $\text{N}{{\text{H}}_{4}}^{+}$ form from the external solution and/or that the higher NRA activity rapidly reduced $\text{N}{{\text{O}}_{3}}^{-}$ to $\text{N}{{\text{H}}_{4}}^{+}.$

Nitrogen nutrition with high levels of $\text{N}{{\text{H}}_{4}}^{+}$ is reported to cause nutrient imbalance as it depresses the uptake of K, Ca and Mg, while increases that of phosphate and sulfate (Roosta and Schjoerring 2007), which was confirmed in our study. The decrease in internal K concentration observed in our study was probably caused by competition with high levels of $\text{N}{{\text{H}}_{4}}^{+}$ (ten Hoopen *et al*. 2010), which in turn may be the cause of the decrease in leaf *ψ*_w_, affecting leaf expansion and DM. Supplementary Ca in plants exposed to high $\text{N}{{\text{H}}_{4}}^{+}$ resulted in increased root Ca and decreased leaf Mg, probably due to the competition between Ca and Mg for uptake sites (Marschner 1995). The nutrient imbalance also explains the more reduced growth of plants with 25 % of N in $\text{N}{{\text{H}}_{4}}^{+}$ compared with plants with 50 % $\text{N}{{\text{H}}_{4}}^{+},$ as a higher root concentration of $\text{N}{{\text{H}}_{4}}^{+}$ directly affected leaf, stem and shoot growth, whereas a higher root $\text{N}{{\text{O}}_{3}}^{-}$ affected root growth.

## Conclusions

The results obtained in the present study indicate that $\text{N}{{\text{H}}_{4}}^{+}$ caused growth reduction, nutrient imbalance, membrane integrity impairment, increased activity of antioxidant enzymes and affected water relations in bell pepper plants; however, as supplementary Ca partially restored the growth of leaves by improving root *L*_o_ and water relations, it may be part of an integral management to enhance the tolerance of important agricultural species to excess $\text{N}{{\text{H}}_{4}}^{+}.$

## Sources of Funding

Our work was funded by Universidad Autónoma Agraria Antonio Narro.

## Contributions by the Authors

E.H.-G. and L.A.V.-A. designed and performed the study, D.L.C. and A.D.C. designed the experiment and wrote the document, and I.A.-T. performed biochemical and statistical analysis.

## Conflict of Interest Statement

None declared.
